# Hemodynamic effects of peri-operative statin therapy in on-pump cardiac surgery patients

**DOI:** 10.1186/1749-8090-7-39

**Published:** 2012-07-13

**Authors:** Jose Hinz, Philipp Gehoff, Hanna Schotola, Morteza Tavakkoli Hosseini, Vassilios N Didilis, Ahmad Fawad Jebran, Anastasia Gehoff, Christoph H Wiese, Egbert Godehard Schulz, Friedrich Albert Schoendube, Aron Frederik Popov

**Affiliations:** 1 Department of Anaesthesiology, Emergency and Intensive Care Medicine, University of Göttingen, Göttingen, Germany; 2 Department of Cardiothoracic Transplantation & Mechanical support, Royal Brompton & Harefield NHS Trust, London, UK; 3 Department of Cardiothoracic Surgery, University Hospital Alexandroupolis, Alexandroupolis, Greece; 4 Department of Thoracic and Cardiovascular Surgery, University of Göttingen, Göttingen, Germany; 5 Institute of Pathology Nordhessen, Kassel, Germany; 6 Center of Nephrology Göttingen, Hypertension Care and Research Unit, Göttingen, Germany; 7 Department of Thoracic and Cardiovascular Surgery, University of Göttingen, Robert-Koch-Straße 40, 37099, Göttingen, Germany

**Keywords:** Cardiac surgery, Statin therapy, Hemodynamic, Outcome

## Abstract

**Background:**

Peri-operative statin therapy in cardiac surgery cases is reported to reduce the rate of mortality, stroke, postoperative atrial fibrillation, and systemic inflammation. Systemic inflammation could affect the hemodynamic parameters and stability. We set out to study the effect of statin therapy on perioperative hemodynamic parameters and its clinical outcome.

**Methods:**

In a single center study from 2006 to 2007, peri-operative hemodynamic parameters of 478 patients, who underwent cardiac surgery with cardiopulmonary bypass, were measured. Patients were divided into those who received perioperative statin therapy (n = 276; statin group) and those who did not receive statin therapy (n = 202; no-statin group). The two groups were compared together using Kolmogorov-Smirnov-Test, Fisher’s-Exact-Test, and Student’s-*T*-test. A p value < 0.05 was considered as significant.

**Results:**

There was no significant difference in the preoperative risk factors. Onset of postoperative atrial fibrillation was not affected by statin therapy. Extended hemodynamic measurements revealed no significant difference between the two groups, apart from Systemic Vascular Resistance Index (SVRI) . The no-statin group had a significantly higher SVRI (882 ± 206 vs. 1050 ± 501 dyn s/cm^5^/m^2^, p = 0.022). Inotropic support was the same in both groups and no significant difference in the mortality rate was noticed. Also, hemodynamic parameters were not affected by different types and doses of statins.

**Conclusions:**

Perioperative statin therapy for patients undergoing on-pump coronary bypass grafting or valvular surgery, does not affect the hemodynamic parameters and its clinical outcome.

## Background

Statins are inhibitors of the 3-hydroxy-3-methylglutaryl-coenzyme A (HMG CoA) reductase. They are an established treatment for hypercholesterolemia by lowering plasma levels of low-density lipid cholesterol (LDL-C) [1,2]. LDL-C plays an important role in the pathogenesis of atherosclerosis [3]. Consequently statins are used effectively in primary and secondary prevention of cardiovascular diseases, like coronary heart disease, myocardial infarction or stroke [4-7]. Apart from their lipid lowering effects, some authors state that patients undergoing cardiac surgery with pulmonary bypass would benefit from intensified perioperative statin therapy. It can reduce the rate of artrial fibrillation, mortality and systemic inflammation [8,9].

Several studies have already demonstrated the pleiotropic effect of the statins. Based on meta-analysis of over 30 000 patients, Liakopoulos et al. confirmed the clinical benefit of preoperative statin therapy in early postoperative adverse outcomes for cardiac surgery patients [10]. They showed a 4.3% absolute risk reduction in artrial fibrillation and a 1.5% absolute risk reduction for early all-cause mortality [10].

Systemic inflammation is a potential complication of cardiopulmonary bypass after cardiac operations [11,12]. Artificial material of extracorporeal circuit induces the activation of blood components and it could result in a systemic inflammatory response syndrome (SIRS) [13]. The surgical trauma could also induce systemic inflammation. Important pro-inflammatory cytokines are mainly Interleukin-6 (IL-6), Interleukin-8 (IL-8) and Tumor necrosis factor-alpha (TNF-α) [14]. Youssef et al. have already shown in mice model, that statins suppress inflammatory reactions in autoimmune diseases [15]. There are clinical studies showing a post-operative decrease of pro-inflammatory cytokines in patients with cardiopulmonary bypass [16,17].

Perioperative statin therapy can influence the postoperative outcome of patients with cardiopulmonary bypass. Meanwhile the effect of statins on peri-operative hemodynamic parameters has not been studied in detail yet. Systemic inflammation induces the release of pro-inflammatory cytokines and the resultant systemic vasodilatation. As a result peripheral resistance decreases and there should be measureable change in hemodynamic parameters. This state is commonly known as hyperdynamic circulation [18]. In view of the fact that statins may have an anti-inflammatory effect, they could potentially improve the postoperative hemodynamics and clinical outcome. The aim of this study was to detect the anti-inflammatory effects of statins by referencing it to the hemodynamic parameters in cardiac surgery patients with cardiopulmonary bypass.

## Methods

The retrospective single centre study was approved by the ethics committee of the Medical Faculty, University of Göttingen, Germany. The study groups were already part of a former prospective study. Individual patient consent was obtained before enrolling in the study. From December 2006 to December 2007, we included 478 patients (312 males and 166 females), who underwent cardiac surgery with cardiopulmonary bypass.

The study population was divided into two groups. One group (276 patients) received perioperative statins and the other group (202 patients) did not.

### Demographic and clinical data

The following demographic and clinical data were collected preoperatively: age, gender, risk factors (body mass index, smoking, hypertension, diabetes mellitus, positive family history, ejection fraction, peripheral arterial disease, neurocerebral events, pulmonary hypertension, chronic obstructive pulmonary disease (COPD), dialysis and renal dysfunction), renal function variables (serum creatinine, serum urea, estimated creatinine clearance and hematocrit), preoperative medications (ß-blockers, ACE inhibitors, oral nitrates, antiarrhythmics, diuretics, antidiabetics, further antihypertensive agents, bronchodilators and anticoagulation), urgency of surgery, associated surgical procedures (coronary artery bypass grafting, valve, combined procedures and others) and the Euroscore [19]. The clinical and demographic characteristics are summarized in Table 1.

**Table 1:** Clinical and demographic characteristics of the study population

	**Statin**	**No Statin**	***p*-value**
	**(n = 276)**	**(n = 202)**	
**Age and gender**			
Age (years)	68 ± 8.9	68 ± 10.9	0.94
Male / Female (%)	69.2/30.8	59.9/40.1	0.03
**Risk factors**			
Body mass index (kg/m²)	28 ± 4.7	27.1 ± 4.4	0.03
Smokers (%)	39.9	27.7	0.006
Hypertension (%)	77.5	68.8	0.03
Diabetes mellitus (%)	37	23.3	0.001
Positive family history (%)	13.4	4	0.11
Ejection fraction (EF; %)	54 ± 14	53 ± 14	0.75
Peripheral disease (%)	8.3	5	0.15
Neurocerebral events (%)	12.3	12.4	0.98
Pulmonary hypertension (%)	5.8	11.9	0.02
COPD (%)	9.1	6.4	0.30
Dialysis (%)	0.4	1	0.39
Renal dysfunction (%)	14.5	13.4	0.73
**Renal function variables**			
Serum creatinine (mg/dl)	1.17 ± 0.75	1.18 ± 0.74	0.82
Urea (mg/dl)	22 ± 13	22 ± 13	0.54
Estimated creatinine clearance (ml/min)	75 ± 32	77 ± 34	0.68
Hematocrit (%)	40 ± 6	39 ± 6	0.31
**Preoperative medications**			
ß-blockers (%)	67	61.4	0.20
ACE inhibitors (%)	60.5	45	0.0008
Oral nitrates (%)	22.5	13.9	0.02
Antiarrhythmics (%)	4	3.5	0.76
Diuretics (%)	40	43.1	0.48
Antidiabetics (%)	21.7	14.9	0.06
Further antihypertensives (%)	25.4	18.3	0.07
Bronchodilator (%)	3.3	3	0.86
Anticoagulation (%)	76.8	59.4	0.0001
**Associated surgical procedures**			
CABG (n = 252 / 52.7%) (%)	68.1	31.7	<0.0001
Valve (n = 107 / 22.4%) (%)	10.9	38.1	<0.0001
Combined procedures (n = 101 / 21.1%) (%)	20.3	22.3	0.65
Other procedures (n = 18 / 3.8%) (%)	0.7	7.9	<0.0001
**Euroscore additive**	5 ± 4	6 ± 4	0.29

Different type and doses of statin preparations and the number of patients are shown in Table 2.

**Table 2:** Statin dosages of the statin group

	n (%)
**Simvastatin**	
10 mg	18 (11)
15 mg	4 (2)
20 mg	64 (39)
25 mg	1 (1)
40 mg	77 (46)
**Atorvastatin**	
10 mg	6 (15)
20 mg	23 (59)
40 mg	8 (21)
80 mg	2 (5)
**Fluvastatin**	
40 mg	5 (21)
80 mg	16 (79)
**Pravastatin**	
5 mg	2 (4)
10 mg	2 (4)
20 mg	15 (30)
40 mg	31 (62)
**Lovastatin**	
20 mg	1 (50)
80 mg	1 (50)

### Perioperative data

The following data were collected from the patient in intensive care unit 24 hours after their cardiac surgery operation: laboratory findings (serum creatinine max., increased in serum creatinie, creatinine clearance min., decrease creatinine clearance, hematocrit min., serum urea max., furosemide mean, serum creatinine phosphokinase (CPK), serum phosphokinase-MB (CPK-MB), potassium, lactat acid levels, usage of renal replacement therapy during hospital stay and leukocytes). To evaluate the severity of acute illness, the RIFLE criteria for acute kidney injury (Risk of renal dysfunction, Injury to the kidney, Failure or Loss of kidney function, and End-stage kidney disease), the Acute Physiology and Chronic Health Evaluation II Score (APACHE II) and the Simplified Acute Physiology Score II (SAPS II) were determined [20-22]. Inotropic support included the need for administration of epinephrine, norepinephrine, enoximone and dobutamine. Also the use of nitroglycerin, amiodarone and cortisone were recorded during the first 24 hours after the operation.

Intraoperative cross-clamp time and cardiopulmonary bypass times were also recorded and the details are summarized in Table 3.

**Table 3:** Perioperative patients’ characteristics within first 24 hours

**Variable**	**Statin**	**No-Statin**	***p-value***
	**(n = 276)**	**(n = 202)**	
***Laboratory findings***			
Creatinine serum max. (mg/dl)	1.9 ± 1.25	2.0 ± 3.8	0.58
Increased in serum creatinine (%)	72 ± 116	76 ± 193	0.78
Creatinine clearance min. (ml/min)	57 ± 32	56 ± 31	087
Decreased creatinine clearance (ml/min)	18 ± 34	20 ± 42	0.72
Hematocrit min. (%)	27 ± 4	27 ± 4	0.83
Urea max. (mg/dl)	39 ± 26	42 ± 57	0.44
Furosemide mean (mg/d)	96 ± 158	79 ± 100	019
S-CPK (U/L)	973 ± 990	1139 ± 2559	0.37
S-CPK-MB (U/L)	45 ± 45	50 ± 77	0.42
Potassium (mmol/L)	4.5 ± 0.5	4.5 ± 0.5	0.48
Lactate (mmol/L)	1.6 ± 1.4	1.6 ± 1.9	0.64
Renal replacement therapy during hospital stay (%)	9.1	8.9	0.95
Leucocytes (10^3^/μl)	15 ± 7	14 ± 8	0.0001
***Scores***			
Rifle Score (1^st^ POD) (n = 285 / 59,6%)			
R (risk) n = 60	32	28	0.72
			I (injury) n = 65	39	26
F (failure) n = 160	88	72			
APACHE II Score	15 ± 6	15 ± 7		0.66	
SAPS II Score	25 ± 7	25 ± 8	0.61		
***Inotropes***					
Epinephrine (mg/d)	9 ± 123	2 ± 8	0.40		
Norepinephrine (mg/d)	2 ± 16	1 ± 6	0.24		
Enoximone (mg/d)	20 ± 114	22 ± 195	0.88		
Dobutamine (mg/d)	9 ± 44	13 ± 69	0.49		
***Other agents***					
Nitroglycerine (mg/d)	12 ± 32	9 ± 21	0.40		
Amiodarone (mg/d)	68 ± 286	60 ± 255	0.73		
Cortisone (mg/d)	21 ± 119	66 ± 449	0.11		
***Operative characteristics***					
Cross-clamp time (min)	94 ± 36	94 ± 41	0.90		
Cardiopulmonary bypass time (min)	147 ± 60	140 ± 78	0.30		

*APACHE* Acute Physiology and Chronic Health Evaluation II Score, *SAPS*: Simplified Acute Physiology Score, *S-CPK* Serum creatinine phosphokinase.

The extended hemodynamic measurements (Table 4) included heart rate, absolute arrhythmia, mean arterial pressure (MAP), central venous pressure (CVP), pulmonary capillary wedge pressure (PCWP), mean pulmonary arterial pressure (PAP_mean_), cardiac index (CI), systemic (SVRI) and pulmonary vascular resistance (PVRI).

**Table 4:** Hemodynamic parameters

**Variable**	**Statin**	**No-Statin**	***p*-value**
	**(n = 276)**	**(n = 202)**	
***Hemodynamic***			
Heart rate (bpm)	82 ± 13	82 ± 12	0.67
MAP (mmHg)	80 ± 8	80 ± 9	0.60
CVP (mmHg)	11 ± 3	11 ± 3	0.205
PCWP (mmHg)	13 ± 5	14 ± 4	0.08
PAP mean (mmHg)	25 ± 5	26 ± 10	0.93
CI (l/min/m^2^)	2.7 ± 0.5	3.2 ± 3.4	0.30
SVRI (dyn∙s∙m^-^²∙cm^-5^)	882 ± 206	1050 ± 501	0.022
PVRI (dyn∙s∙m^-^²∙cm^-5^)	190 ± 121	247 ± 208	0.10

*MAP* Mean arterial pressure, *CVP* central venous pressure, *PCWP* Pulmonary capillary wedge pressure, *PAP* Mean pulmonary arterial pressure, *CI* Cardiac index, *SVRI* Systemic Vascular Resistance Index *PVRI* Pulmonary Vascular Resistance Index.

### Postoperative data

To characterize postoperative outcomes of our patients, we collected the following values: red blood cell transfusion, fresh frozen plasma transfusion, prothrombin complex concentrates usage (PCC), intra-aortic balloon pump (IABP), extracorporeal membrane oxygenation (ECMO), length of ICU stay, hospital stay and in-hospital death. In-hospital mortality was defined as all-cause mortality (Table 5).

**Table 5:** Postoperative outcomes of patients

**Variable**	**Statin**	**No-Statin**	***p*-value**
	**(n = 276)**	**(n = 202)**	
***Postoperativem outcomes of patients***			
Red blood cells transfused (ml/d)	250 ± 492	271 ± 750	0.70
Fresh frozen plasma (ml/d)	53 ± 317	113 ± 750	0.23
PCC (U/d)	3.6 ± 60	19.8 ± 204	0.21
IABP (%)	9.8	5	0.05
ECMO (%)	0.4	0	0.50
Onset of atrial fibrillation (%)	18	16	0.51
Length of ICU stay (d)	7 ± 15	7 ± 12	0.99
Hospital stay (d)	25 ± 20	24 ± 21	0.71
***Mortality***			
In-hospital death (n = 38 / 7.9%) (%)	9.1	6.4	0.29

*PCC* Prothrombin complex concentrates, *IABP* Intra-aortic balloon pump, *ECMO* Extracorporeal membrane oxygenation, *ICU* Intensive care unit.

### Statistical analysis

All statistical analyses were performed using Statistica 9.0, StatSoft, Hamburg, Germany. To test for normal distribution Kolmogorov-Smirnov-Test was used. Ordinal scaled variables are presented as mean ± standard deviation (SD) and categorical variables are presented as absolute numbers or percentage. Comparisons of categorical scaled variables for patients with and without preoperative statin therapy were made with Fisher’s-Exact-Test and ordinal variables were compared with Student’s-*T*-test. A p value < 0.05 was considered as statistically significant. A multifactorial analysis was also performed to detect any statistical difference while comparing different types and/or doses of statins.

## Results

### Clinical and demographic data

There was no difference between patients in the statin group and the no statin group concerning average age. Both groups had more male than female patients (p = 0.03). Statin groups had higher rates of Body Mass Index (p = 0.03), Cigarette Smoking (p = 0.006), Hypertension (p = 0.03) and Diabetes Mellitus (p = 0.001). There was a higher rate of pulmonary hypertension in the no statin group (p = 0.02). Other risk factors and renal function parameters showed no differences between the two groups. The use of ACE inhibitors (p = 0.0008), oral nitrates (p = 0.02) and anticoagulation (p = 0.0001) was higher in the statin group. More patients of the statin group underwent coronary artery bypass grafting (p < 0.0001), while the no statin group had more valve surgery (p < 0.0001) and other procedures (p < 0.0001). The Euroscore revealed no significant differences between the two groups. All data are summarised in Table 1.

### Statin drugs and dosages

In a multivariate analysis, the different types and doses of statin drugs did not affect the perioperative mortality in our study groups (Table 2).

### Perioperative data

Increased leucocytes were measured in the statin group (p = 0.0001). The groups did not differ in SAPS II Score, APACHE II Score and RIFLE Score. The usage of inotropic support achieved no statistical significance (Table 3). Extended hemodynamic measurements revealed no significant difference between the two groups, apart from SVRI (Systemic Vascular Resistance Index). The no-statin group had a significantly higher SVRI (882 ± 206 vs. 1050 ± 501 dyn*s*cm^-5^*m^-2^; p = 0.022). The results of the hemodynamic parameters are shown in Table 4.

### Postoperative data

There was no difference in blood and fresh plasma transfusion, as well as in dose of Prothrombin Complex Concentrate (PCC) among the two groups. Also, there was no statistical difference in length of stay in the intensive care unit, hospital stay and mortality (Table 5).

## Discussion

The aim of this study was to check the proclaimed anti-inflammatory effects of statins on postoperative hemodynamic parameters. We did not detect a clinical impact of perioperative statin therapy in patients undergoing cardiac surgery with cardiopulmonary bypass.

The inflammatory response after cardiopulmonary bypass is thought to have significant role regarding complications following cardiopulmonary bypass in cardiac surgery patients [11,12]. It was shown that the pleiotropic effect of statins have a clinical benefit regarding atrial fibrillation and all-cause mortality [10] due to their anti-inflammatory effect. The release of pro-inflammatory cytokines (IL-6, IL-8, and TNF-α) can induce a systemic inflammation which can lead to a change of the vascular resistance. Statin therapy is being recognized to have anti-inflammatory effect of injury after cardiopulmonary bypass; however there are no clinical data which confirm the influence of statin therapy on perioperative hemodynamics.

In our study SVRI in the statin group was significantly lower. The other ascertained hemodynamic parameters revealed no significant differences between the two groups. A decrease of SVRI is an evidence for systemic inflammation, so we could not find an evidence for the anti-inflammatory effect of statins. Also leucocytes were increased in both groups, while being higher in the statin group. We suppose that increased ACE inhibitor treatment in the statin group might have influenced our study outcome. Boeken et al. pointed out that long-term treatment with ACE inhibitors caused vasodilatatory effects and enhanced perioperative need for catecholamines. Furthermore the ACE inhibitor induced vasodilatation could be one reason for SIRS [23]. So our decreased SVRI and increased leukocytes probably arose from long-term ACE inhibitor treatment. This not surprising because, the majority of cases in the no statin group with lower cholesterol levels underwent valve surgery. 60.5% of the patients in the statin group took ACE inhibitors, because the clinical impact of ACE inhibitor therapy for patients with coronary heart disease is already approved [24]. But their use in valve surgery patients is still, restricted due to their afterload lowering effects.

Further assumed pleiotropic statin effects are prevention of postoperative artrial fibrillation and mortality. In our study onset of artrial fibrillation was unaffected by statin therapy. Also mortality was quiet uniform across all patients. In 2010 Boerger et al. [25] found similar results in patients undergoing isolated valve surgery. They failed to detect a protective effect of preoperative statin therapy on perioperative outcomes or long-term survival. These results raise the question about the pleiotropic effect of statins, the clinical impact of perioperative statin therapy remains unclear.

## Limitations

Several limitations of this study merit attention. The primary limitations to this study are its retrospective design and the analysis of patients from a single institution and therefore conclusions are necessarily limited in their application.

It does not seem to be very surprising that the statin group is dominated by Coronary artery bypass graft patients, whereas the no-statin group consists of patients with valvular heart disease with or without coincident coronary disease. This can have potential influence on perioperative hemodynamic data. In this retrospective analysis we did not measure the proclaimed inflammatory effect of on-pump cardiac surgery in terms of circulating markers of inflammation like IL-6, IL-8, and TNF-α. However, clinical outcome analysis was focused on perioperative hemodynamics and we are not able to provide information on late complications, quality of life, and cause of death. Moreover, the patient cohort underwent administration of multiple statin drugs at different dosages. However, to our knowledge this is the first study who investigates the perioperative hemodynamic in terms of statin treatment in a large cohort of cardiac surgery patients.

## Conclusions

The data of this study do not support a benefit in perioperative statin therapy for patients undergoing on-pump cardiac surgery, in terms of postoperative hemodynamic improvement or clinical outcome.

## Abbreviations

ACE = Angiotensin converting enzyme; APACHE II = Acute Physiology and Chronic Health Evaluation II Score; BMI = Body mass index; CABG = Coronary artery bypass graft; CI = Cardiac index; COPD = Chronic obstructive pulmonary disease; CPK = Creatinine phosphokinase; CVP = Central venous pressure; ECMO = Extracorporeal membrane oxygenation; HMG CoA = 3-hydroxy-3-methylglutaryl-coenzyme A; IABP = Intra-aortic balloon pump; ICU = Intensive care unit; IL = Interleukin; LDL-C = Low-density lipid cholesterol; MAP = Mean arterial pressure; PAPmean = Mean pulmonary arterial pressure; PCC = Prothrombin Complex Concentrate: combination of blood clotting factors II, VII, IX and X, as well as protein C and S; PCWP = Pulmonary capillary wedge pressure; PVRI = Pulmonary vascular resistance index; RIFLE = Risk of renal dysfunction, Injury to the kidney, Failure or Loss of kidney function, and End-stage kidney disease; SAPS II = Simplified Acute Physiology Score II; SD = Standard deviation; SIRS = Systemic inflammatory response syndrome; SVRI = Systemic Vascular Resistance Index; TNF-α = Tumor necrosis factor-alpha.

## Misc

Jose Hinz and Philipp Gehoff authors contributed equally to this manuscript.

## Competing interests

The authors declare that they have no competing interests.

## Authors' contributions

JH and PG wrote the paper and have made substantial contributions to conception and design. HS, AG, MTH, VND, and CHW have made substantial contributions to analysis of data. AFJ, EGS have been involved in drafting the manuscript. FAS have made important comments. AFP has been involved in revising it critically for important intellectual content and co-wrote the paper. All authors read and approved the final manuscript.
